# Supplementary material for: Efficacy of Clostridium butyricum Supplementation Combined with Phototherapy for Neonatal Hyperbilirubinemia: A Systematic Review and Meta-Analysis
Source: Microorganisms. 2025 Jun 20;13(7):1441. doi: 10.3390/microorganisms13071441 (PMC12300382; doi:10.3390/microorganisms13071441)
Supplement: Supplementary file 1 [file microorganisms-13-01441-s001.zip › microorganisms-3706894-supplementary/Supplementary File 2. Search strategy for each database.docx]

**Supplementary File S2**. Search strategy used in each database.

Medline via PubMed

|  | Searches | Results |
| --- | --- | --- |
| #1 | (infant, newborn[MeSH] OR newborn OR neonate OR neonatal OR premature OR low birth weight OR VLBW OR LBW or infan* or neonat*) | 322,327 |
| #2 | jaundice[MeSH Terms]) OR (hyperbilirubinaemia[MeSH Terms]) OR (jaundice[Title/Abstract]) OR (hyperbilirubinaemia[Title/Abstract]) OR jaundice OR hyperbilirubinaemia | 8,443 |
| #3 | probiotic[MeSH Terms] OR probiotics[MeSH Terms] OR pro biotic*[Title/Abstract] OR probiotic*[Title/Abstract] OR Lactobacillus OR Bifidobacterium OR S. boulardii OR yeast OR yogurt | 92,245 |
| #4 | randomized controlled trial [pt] OR controlled clinical trial [pt] OR randomized [tiab] OR placebo [tiab] OR drug therapy [sh] OR randomly [tiab] OR trial [tiab] OR groups [tiab] NOT (animals [mh] NOT humans [mh]) | 1,508,439 |
| #5 | #1 AND #2 AND #3 AND #4 | **24** |

EMBASE

|  | Searches | Results |
| --- | --- | --- |
| #1 | 'infant'/exp OR infant:ti,ab OR 'newborn'/exp OR newborn:ti,ab OR 'neonate'/exp OR neonate:ti,ab OR 'premature'/exp OR premature:ti,ab | 1,688,326 |
| #2 | ‘jaundice'/exp OR jaundice:ti,ab OR 'hyperbilirubinemia'/exp OR hyperbilirubinemia:ti,ab | 120,914 |
| #3 | probiotic*:ti,ab OR Lactobacillus:ti,ab OR Bifidobacterium:ti,ab OR Boulardii:ti,ab OR yeast:ti,ab OR yogurt:ti,ab | 102,827 |
| #4 | (human not animal) AND (randomized controlled trial or controlled clinical trial or randomized or placebo or clinical trials as topic or randomly or trial or clinical trial) | 2,610,091 |
| #5 | #1 AND #2 AND #3 AND #4 | 37 |
| #6 | #5 AND (2020:py OR 2021:py OR 2022:py OR 2023:py OR 2024:py OR 2025:py) | **19** |

CENTRAL

|  | Searches | Results |
| --- | --- | --- |
| #1 | MeSH descriptor: [Infant, Newborn] explode all trees | 23,501 |
| #2 | MeSH descriptor: [Jaundice] explode all trees | 259 |
| #3 | MeSH descriptor: [Probiotics] explode all trees | 3,625 |
| #4 | (probiotics):ti,ab,kw OR (probiotic*):ti,ab,kw OR (Lactobacillus):ti,ab,kw OR (Bifidobacterium):ti,ab,kw OR (Boulardii):ti,ab,kw OR (yeast):ti,ab,kw OR (yogurt):ti,ab,kw | 15,449 |
| #5 | (#1 AND #2) AND (#3 OR #4) | **2** |

CNKI

| #1 | (SU=('新生儿'+'infants'+'neonates') AND SU=('jaundice'+'hyperbilirubinemia') AND SU =('益生菌'+'乳酸菌'+'双歧杆菌'+'乳杆菌'+'链球菌'+'肠球菌'+'芽孢杆菌'+'枯草杆菌'+'酪酸梭菌'+'酵母菌'+'比菲德氏菌'+'probiotic'+'Bifidobacterium'+'Lactobacillus'+'Streptococcus'+'Enterococcus'+'Bacillus'+'Clostridium butyricum'+'Saccharomyces boulardii') | 394 |
| --- | --- | --- |

Wan Fang Database

| #1 | 主题:(益生菌 or 乳酸菌 or 双歧杆菌 or 乳杆菌 or 链球菌 or 肠球菌 or 芽孢杆菌 or 枯草杆菌 or 酪酸梭菌 or 酵母菌 or 比菲德氏菌 or probiotic or Bifidobacterium or Lactobacillus or Streptococcus or Enterococcus or Bacillus or Clostridium butyricum or Saccharomyces boulardii) | 223 |
| --- | --- | --- |

Chinese Scientific Journal Database (VIP)

| #1 | M=(新生儿 or infants or neonates) AND M=(jaundice or hyperbilirubinemia) AND M=(益生菌 or 乳酸菌 or 双歧杆菌 or 乳杆菌 or 链球菌 or 肠球菌 or 芽孢杆菌 or 枯草杆菌 or 酪酸梭菌 or 酵母菌 or 比菲德氏菌 or probiotic or Bifidobacterium or Lactobacillus or Streptococcus or Enterococcus or Bacillus or Clostridium butyricum or Saccharomyces boulardii) | 165 |
| --- | --- | --- |

Oriental Medicine Advanced Searching Integrated System (OASIS)

| #1 | 신생아황달 | 1 |
| --- | --- | --- |

Korean studies Information Service System (KISS)

| #1 | 신생아황달 | 37 |
| --- | --- | --- |

Korea Citation Index (KCI)

| #1 | 신생아황달 | 53 |
| --- | --- | --- |

Research Information Sharing Service (RISS)

| #1 | 신생아황달 | 133 |
| --- | --- | --- |

Korean Medical database (KMbase)

| #1 | 황달 | 3 |
| --- | --- | --- |
