# Supplementary material for: Efficacy of Clostridium butyricum Supplementation Combined with Phototherapy for Neonatal Hyperbilirubinemia: A Systematic Review and Meta-Analysis
Source: Microorganisms. 2025 Jun 20;13(7):1441. doi: 10.3390/microorganisms13071441 (PMC12300382; doi:10.3390/microorganisms13071441)
Supplement: Supplementary file 1 [file microorganisms-13-01441-s001.zip › microorganisms-3706894-supplementary/Supplementary File 3. Adverse events.docx]

**Supplementary File S3**. Adverse events

| First author (year) | Group | N (Total) | N (Adverse events) | Details |
| --- | --- | --- | --- | --- |
| Gao (2021) [14] | E | 50 | 5 | fever (n=2), rash (n=1), diarrhea (n=1), dehydration (n=1) |
|  | C | 50 | 14 | fever (n=6), rash (n=4), diarrhea (n=3), dehydration (n=1) |
| Huang (2023) [15] | E | 32 | 3 | diarrhea (n=0), vomiting (n=1), rash (n=1), loss of appetite (n=1) |
|  | C | 32 | 4 | diarrhea (n=1), vomiting (n=1), rash (n=1), loss of appetite (n=1) |
| Lai (2020) [16] | E | 50 | 5 | fever (n=2), diarrhea (n=1), vomiting (n=1), rash (n=1) |
|  | C | 50 | 4 | fever (n=1), diarrhea (n=1), vomiting (n=1), rash (n=1) |
| Li (2020a) [17] | E | 46 | 1 | rash (n=1) |
|  | C | 46 | 1 | diarrhea (n=1) |
| Li (2024) [19] | E | 49 | 0 | No significant adverse events were observed. |
|  | C | 49 | 0 | No significant adverse events were observed. |
| Lin (2022) [20] | E | 58 | 3 | No detailed information regarding adverse event. |
|  | C | 58 | 7 | No detailed information regarding adverse event. |
| Liu (2024) [21] | E | 90 | 2 | rash (n=1), dry skin (n=1), diarrhea (n=0) |
|  | C | 90 | 7 | rash (n=3), dry skin (n=2), diarrhea (n=2) |
| Ren (2020) [22] | E | 30 | 1 | rash (n=1), fever (n=0), diarrhea (n=0) |
|  | C | 30 | 8 | rash (n=3), fever (n=2), diarrhea (n=3) |
| Shi (2022) [23] | E | 43 | 4 | fever (n=2), diarrhea (n=1), vomiting (n=1), rash (n=1) |
|  | C | 43 | 5 | fever (n=1), diarrhea (n=1), vomiting (n=2), rash (n=1) |
| Song (2024) [24] | E | 45 | 4 | fever (n=2), diarrhea (n=0), vomiting (n=1), rash (n=1) |
|  | C | 45 | 6 | fever (n=1), diarrhea (n=2), vomiting (n=2), rash (n=1) |
| Sun (2024) [25] | E | 38 | 2 | vomiting (n=0), diarrhea (n=0), rash (n=1), fever (n=1) |
|  | C | 38 | 5 | vomiting (n=2), diarrhea (n=1), rash (n=2), fever (n=0) |
| Wang (2023a) [26] | E | 30 | 3 | dehydration (n=1), hypoglycemia(n=1), rash (n=1), bronze baby syndrome (n=0) |
|  | C | 30 | 4 | dehydration (n=1), hypoglycemia(n=1), rash (n=1), bronze baby syndrome (n=1) |
| Wang (2023b) [27] | E | 48 | 2 | vomiting (n=0), fever (n=1), allergy (n=0), diarrhea (n=1), loss of appetite (n=0) |
|  | C | 48 | 9 | vomiting (n=2), fever (n=1), allergy (n=1), diarrhea (n=3), loss of appetite (n=2) |
| Wang (2023c) [28] | E | 39 | 2 | abdominal discomfort (n=1), allergy (n=0), digestive problems (n=1) |
|  | C | 38 | 2 | abdominal discomfort (n=1), allergy (n=0), digestive problems (n=1) |
| Xiong (2020) [29] | E | 44 | 1 | fever (n=1), diarrhea (n=0), loss of appetite (n=0) |
|  | C | 44 | 8 | fever (n=3), diarrhea (n=3), loss of appetite (n=2) |
| Zhao (2020) [30] | E | 31 | 2 | loss of appetite (n=0), rash (n=1), diarrhea (n=1) |
|  | C | 31 | 4 | loss of appetite (n=1), rash (n=1), diarrhea (n=2) |
| Zhang (2023) [31] | E | 37 | 3 | diarrhea (n=1), constipation (n=1), rash (n=1), low-grade fever (n=0) |
|  | C | 37 | 10 | diarrhea (n=4), constipation (n=2), rash (n=3), low-grade fever (n=1) |
| Zhang (2024) [32] | E | 39 | 1 | diarrhea (n=1), digestive problems (n=0), vomiting (n=0), fever (n=0) |
|  | C | 39 | 9 | diarrhea (n=3), digestive problems (n=2), vomiting (n=2), fever (n=2) |
| Zhu (2022) [33] | E | 69 | 7 | fever (n=4), vomiting (n=2),diarrhea (n=1) |
|  | C | 71 | 18 | fever (n=11), vomiting (n=3),diarrhea (n=4) |

E, experimental group; C, control group; NR, not reported
