# Supplementary material for: Efficacy of Clostridium butyricum Supplementation Combined with Phototherapy for Neonatal Hyperbilirubinemia: A Systematic Review and Meta-Analysis
Source: Microorganisms. 2025 Jun 20;13(7):1441. doi: 10.3390/microorganisms13071441 (PMC12300382; doi:10.3390/microorganisms13071441)
Supplement: Supplementary file 1 [file microorganisms-13-01441-s001.zip › microorganisms-3706894-supplementary/Supplementary File 4. Outcome measurement & Result (p -value).docx]

**Supplementary File S4**. Outcome measurement & Result(*p* -value)

| First author (year) | Outcome measurement & Result(*p* -value) |
| --- | --- |
|  |  |
| Gao (2021) [14] | (1) Total effective rate: E>C ^a^  (2) Adverse events: E<C ^a^  (3) Transcutaneous Bilirubin level: E<C ^a^(72h after treatment) |
|  |  |
| Huang (2023) [15] | (1) Total effective rate: E>C ^a^  (2) Adverse events: E>C ^b^  (3) Serum bilirubin level  1) TBiL: E<C ^a^  2) DBiL: E<C ^a^  3) IBiL: E<C ^a^  (4) Growth Parameters(72h after treatment)  1) length: E>C ^a^  2) weight: E>C ^a^  3) head circumference: E>C ^a^  (5) Apolipoprotein M: E<C ^a^  (6) Neuron-specific enolase: E<C ^a^  (7) C-reactive protein: E<C ^a^ |
|  |  |
| Lai (2020) [16] | (1) Total effective rate: E>C ^a^  (2) Adverse events: E>C ^b^  (3) Transcutaneous Bilirubin level: E<C ^a^  (4) The time of jaundice fading (d) : E<C ^a^ |
|  |  |
|  |  |
|  |  |
| Li (2020a) [17] | (1) Total effective rate: E>C ^a^  (2) Adverse events: E=C ^b^  (3) Serum bilirubin level  1) TBiL: E<C ^a^  2) DBiL: E<C ^a^  3) IBiL: E<C ^a^ |
|  |  |
| Li (2020b) [18] | (1) Total effective rate: E>C ^a^  (2) Serum bilirubin level (after treatment)  1) TBiL: E<C ^a^  2) IBiL: E<C ^a^ |
|  |  |
|  |  |
| Li (2024) [19] | (1) Total effective rate: E>C ^a^  (2) Adverse events: E<C ^a^  (3) Serum bilirubin level  1) TBiL: E<C ^c^  2) IBiL: E<C ^c^  (4) Neuron-specific enolase: E<C ^c^  (5) S100B protein: E<C ^c^  (6) Glial cell line-derived neurotrophic factor: E<C ^c^ |
|  |  |
| Lin (2022) [20] | (1) Total effective rate: E>C ^a^  (2) Adverse events: E<C ^a^  (3) Serum bilirubin level  1) TBiL: E<C ^a^ (after treatment)  (4) C-reactive protein: E<C ^a^  (5) Alanine aminotransferase: E<C ^a^  (6) Aspartate aminotransferase: E<C ^a^  (7) Serum albumin: E<C ^a^  (8) Length of hospital stays: E<C ^a^ |
|  |  |
| Liu (2024) [21] | (1) Total effective rate: E>C ^a^  (2) Adverse events  (3) Transcutaneous Bilirubin level: E<C ^c^  (4) Serum bilirubin level  1) TBiL: E<C ^c^  2) DBiL: E<C ^c^  (5) The time of jaundice fading(d): E<C ^c^  (6) Length of hospital stays(d): E<C ^c^  (7) Time to meconium passage(d): E<C ^c^  (8) Duration of phototherapy(h): E<C ^c^ |
|  |  |
| Ren (2020) [22] | (1) Adverse events: E<C ^a^  (2) Serum bilirubin level  1) TBiL: E<C ^c^  2) IBiL: E<C ^c^ |
|  |  |
| Shi (2022) [23] | (1) Total effective rate: E<C ^a^  (2) Adverse events: E<C ^b^  (3) Serum bilirubin level  1) TBiL: E<C ^c^  2) DBiL: E<C ^c^  3) IBiL: E<C ^c^  (4) Serum immunoglobulin levels (Immunoglobulin A, Immunoglobulin G, Immunoglobulin M)  1) Immunoglobulin A: E>C ^c^  2) Immunoglobulin G: E>C ^c^  3) Immunoglobulin M: E>C ^c^ |
|  |  |
|  |  |
|  |  |
| Song (2024) [24] | (1) Total effective rate: E>C ^a^  (2) Adverse events: E<C ^b^  (3) Serum bilirubin level  1) TBiL: E<C ^c^  2) DBiL: E<C ^c^  3) IBiL: E<C ^c^  (4) C-reactive protein: E<C ^c^  (5) Procalcitonin: E<C ^c^  (6) Interleukin-6: E<C ^c^ |
|  |  |
| Sun (2024) [25] | (1) Adverse events: E<C ^b^  (2) Serum bilirubin level  1) TBiL: E<C ^c^  2) DBiL: E<C ^c^  3) IBiL: E<C ^c^  (3) The time of jaundice fading(d) : E<C ^c^  (4) Length of hospital stays: E<C ^a^  (5) Stool frequency (times/d): E>C ^c^  (6) Duration of phototherapy(d) : E<C ^c^ |
|  |  |
| Wang (2023a) [26] | (1) Total effective rate: E>C ^a^  (2) Adverse events: E<C ^b^  (3) Transcutaneous Bilirubin level: E<C ^b^  (4) Serum bilirubin level  1) TBiL: E<C ^a^  2) IBiL: E<C ^c^ |
|  |  |
|  |  |
|  |  |
| Wang (2023b) [27] | (1) Total effective rate: E>C ^a^  (2) Adverse events: E<C ^a^  (3) Serum immunoglobulin levels (Immunoglobulin A, Immunoglobulin G, Immunoglobulin M)  1) Immunoglobulin A: E>C ^c^  2) Immunoglobulin G: E>C ^c^  3) Immunoglobulin M: E>C ^c^  (4) Neonatal Behavioral Neurological Assessment score: E>C ^a^  (5) Beta-2 Microglobulin : E<C ^a^  (6) Gamma-Glutamyl Transferase: E<C ^a^  (7) 25-Hydroxyvitamin D₃: E>C ^a^  (8) Lactate Dehydrogenase: E<C ^a^  (9) Cystatin C: E<C ^a^ |
| Wang (2023c) [28] | (1) Total effective rate: E>C ^a^  (2) Adverse events: E<C ^b^  (3) Serum T lymphocyte:  1) CD4⁺ / CD8⁺: E>C ^a^  (4) Intestinal flora  *Bifidobacterium*: E>C ^a^  *Lactobacillus*: E>C ^a^  *Enterobacteriaceae*: E<C ^a^  *Enterococcus*: E<C ^a^ |
| Xiong (2020) [29] | (1) Total effective rate: E>C ^a^  (2) Adverse events: E<C ^a^  (3) Serum bilirubin level  1) TBiL: E<C ^a^  2) DBiL: E<C ^a^  3) IBiL: E<C ^a^ |
| Zhao (2020) [30] | (1) Total effective rate: E>C ^a^  (2) Adverse events: E<C ^b^  (3) Transcutaneous Bilirubin level: E<C ^a^  (4) Serum bilirubin level  1) TBiL: E<C ^c^  (5) The time of jaundice fading (d) : E<C ^c^  (6) Serum immunoglobulin levels (Immunoglobulin A, Immunoglobulin G, Immunoglobulin M)  1) Immunoglobulin A: E>C ^a^  2) Immunoglobulin G: E>C ^a^  3) Immunoglobulin M: E>C ^a^  (7) Neonatal Behavioral Neurological Assessment score: E>C ^a^ |
| Zhang (2023) [31] | (1) Total effective rate: E>C ^b^  (2) Adverse events: E<C ^a^  (3) Serum bilirubin level  Duration until bilirubin levels returned to normal (d) : E<C ^c^  (4) C-reactive protein: E<C ^a^  (5) Length of hospital stays : E<C ^c^  (6) Duration of phototherapy (d) : E<C ^c^  (7) Procalcitonin: E<C ^a^  (8) Interleukin-6: E<C ^c^  (9) Serum T lymphocyte  1) CD4⁺ / CD8⁺: E>C ^a^ |
| Zhang (2024) [32] | (1) Total effective rate: E>C ^a^  (2) Adverse events: E<C ^a^  (3) Serum bilirubin level  1) Average daily decrease in bilirubin level: E>C ^c^  2) Bilirubin: E<C ^c^  (4) The time of jaundice fading (d) : E<C ^c^ |
| Zhu (2022) [33] | (1) Total effective rate: E>C ^a^  (2) Adverse events: E<C ^a^  (3) Serum bilirubin level  1) TBiL: E<C ^c^  2) DBiL: E<C ^c^  3) IBiL: E<C ^c^  4) Average daily decrease in bilirubin level: E>C ^c^  (5) The time of jaundice fading (d) : E<C ^c^ |
|  |  |
|  |  |

^a^, *p* < 0.05; ^b^, *p* > 0.05; ^c^, *p* < 0.001; d, day; TBiL, Total Bilirubin; DBiL, Direct Bilirubin; IBiL, Indirect Bilirubin; CD, Cluster of Differentiation
