# Supplementary material for: Efficacy of Clostridium butyricum Supplementation Combined with Phototherapy for Neonatal Hyperbilirubinemia: A Systematic Review and Meta-Analysis
Source: Microorganisms. 2025 Jun 20;13(7):1441. doi: 10.3390/microorganisms13071441 (PMC12300382; doi:10.3390/microorganisms13071441)
Supplement: Supplementary file 1 [file microorganisms-13-01441-s001.zip › microorganisms-3706894-supplementary/Supplementary File 5. Sensitivity analysis.docx]

**Supplementary File S5.** Sensitivity analysis for the total bilirubin levels

| Excluding study | SMD | 95%CI | I^2^ (%) | *p-*value |
| --- | --- | --- | --- | --- |
| Huang 2023 | -1.31 | -1.95, 0.68 | 94 | <0.00001 |
| Li 2020a | -1.68 | -2.42, -0.95 | 95 | <0.00001 |
| Li 2024 | -1.57 | -2.33, -0.80 | 95 | <0.00001 |
| Liu 2024 | -1.61 | -2.42, -0.80 | 95 | <0.00001 |
| Ren 2020 | -1.62 | -2.37, -0.88 | 95 | <0.00001 |
| Song 2024 | -1.24 | -1.80, -0.67 | 92 | <0. 0001 |
| Sun 2024 | -1.38 | -2.05, -0.72 | 94 | <0.00001 |
| Wang 2023a | -1.63 | -2.37, -0.88 | 95 | <0.00001 |
| Xiong 2020 | -1.69 | -2.42, -0.95 | 95 | <0.00001 |
| Zao 2020 | -1.65 | -2.39, -0.91 | 95 | <0.00001 |

SMD, standardized mean difference; CI, confidence interval
